# Colonic Lipoma as a Lead Point for Intussusception in an Adult

**DOI:** 10.7759/cureus.82286

**Published:** 2025-04-15

**Authors:** Hasham Ramzan, Tarun Jain

**Affiliations:** 1 Medical Imaging, North Canberra Hospital, Canberra, AUS; 2 Radiology, North Canberra Hospital, Canberra, AUS

**Keywords:** abdominal pain, general surgery, histology, intestinal obstruction, intussusception, lipoma

## Abstract

We present a case of a 38-year-old female with a large colonic lipoma leading to colocolic intussusception. The patient presented with severe intermittent abdominal pain and was diagnosed through ultrasound and CT imaging. Surgical intervention was required to resolve the intussusception and remove the lipoma. Histological examination confirmed the diagnosis of lipoma. Although it is rare for intussusception to occur in adults, this case demonstrates how it may present.

## Introduction

Intussusception is a rare condition where one part of the intestine slides into another, causing an obstruction. Although it is more frequently seen in children, adult intussusception is uncommon and typically linked to a pathological lead point such as a tumor, polyp, or adhesion [1]. The incidence rate in adults ranges from 1 in 20,000 to 1 in 100,000 [2].

Colonic lipomas are benign tumors composed of mature adipose tissue and can occasionally cause symptoms if they lead to intussusception [3]. To confirm the diagnosis of intussusception caused by a colonic lipoma, a colonic biopsy is usually necessary. This avoids misdiagnosing inflammatory changes surrounding the lipoma as an inflammatory bowel disease [3].

This case report aims to explain how colonic intussusception may present and what the typical radiological findings are on both ultrasound and computed tomography (CT) imaging.

## Case presentation

A 38-year-old female presented to the hospital's Emergency Department with severe intermittent periumbilical pain. The pain was crampy, worsened after eating and drinking, and had been ongoing for eight days. The patient had no significant past medical history but reported anxiety, post-traumatic stress disorder, and iron deficiency anemia.

**Figure 1:**
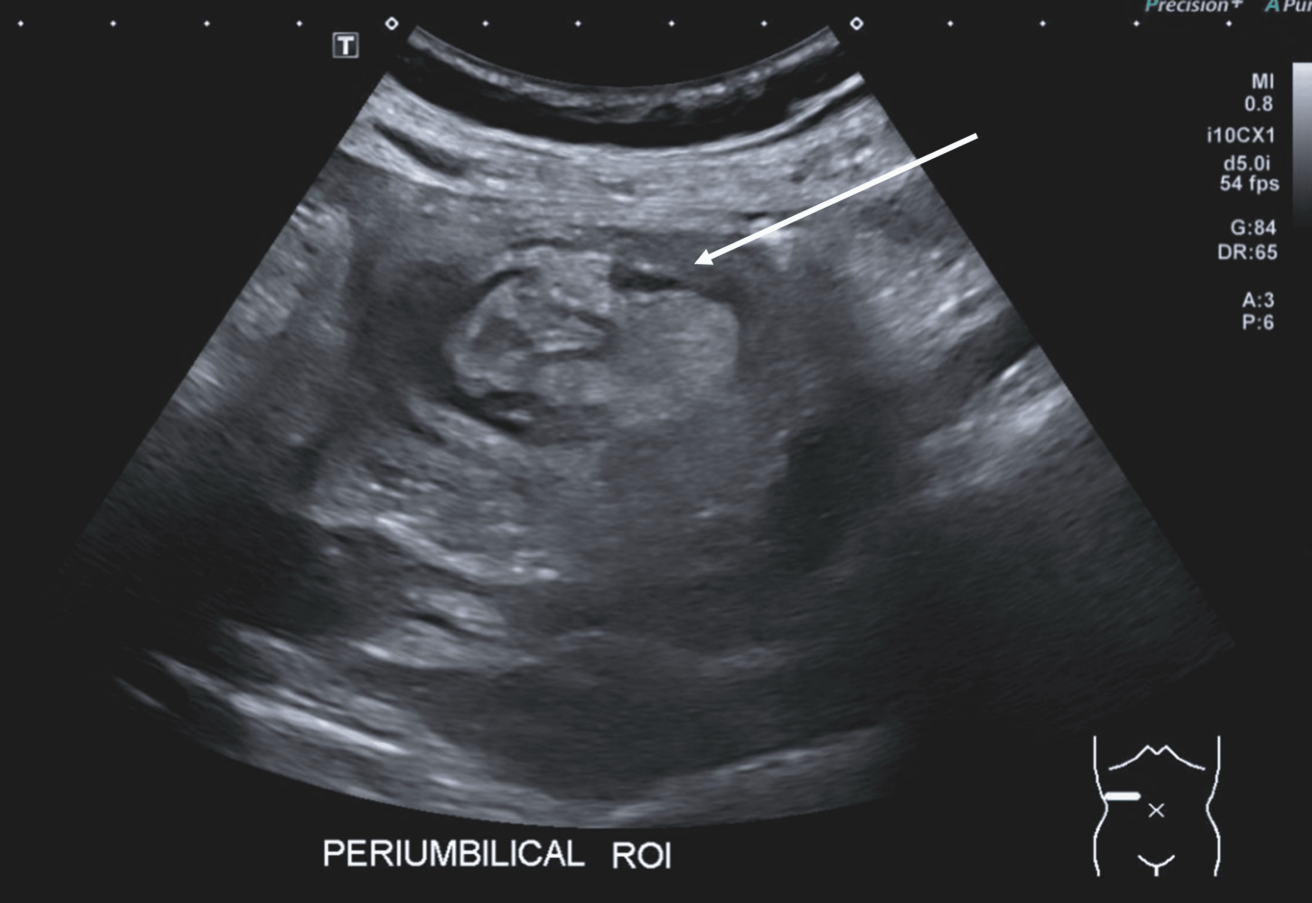
Ultrasound showing the characteristic donut sign seen in intussusception This is characterized by concentric alternating echogenic (bright) and hypoechoic (dark) bands; these bands are formed by the layers of the intestine involved in the intussusception.

On examination, the patient was tender to palpation in the suprapubic and periumbilical regions but had a soft abdomen. Initial blood tests were unremarkable. An ultrasound scan suggested intussusception in the right periumbilical location (Figure 1). 

**Figure 2:**
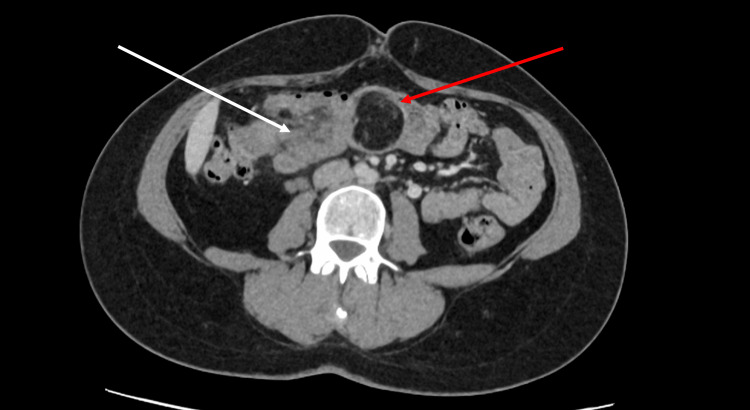
Axial slice CT scan with contrast in portal venous phase, shows colonic intussusception with lipoma as the lead point Red arrow - Shows the appearance of the lipoma, with a darker (hypodense) structure compared to the surrounding tissue. White arrow - Indicates the appearance of intussusception on CT imaging in axial view. You can see the portion of the colon that has slid into the adjacent section of the colon that is enveloping it.

**Figure 3:**
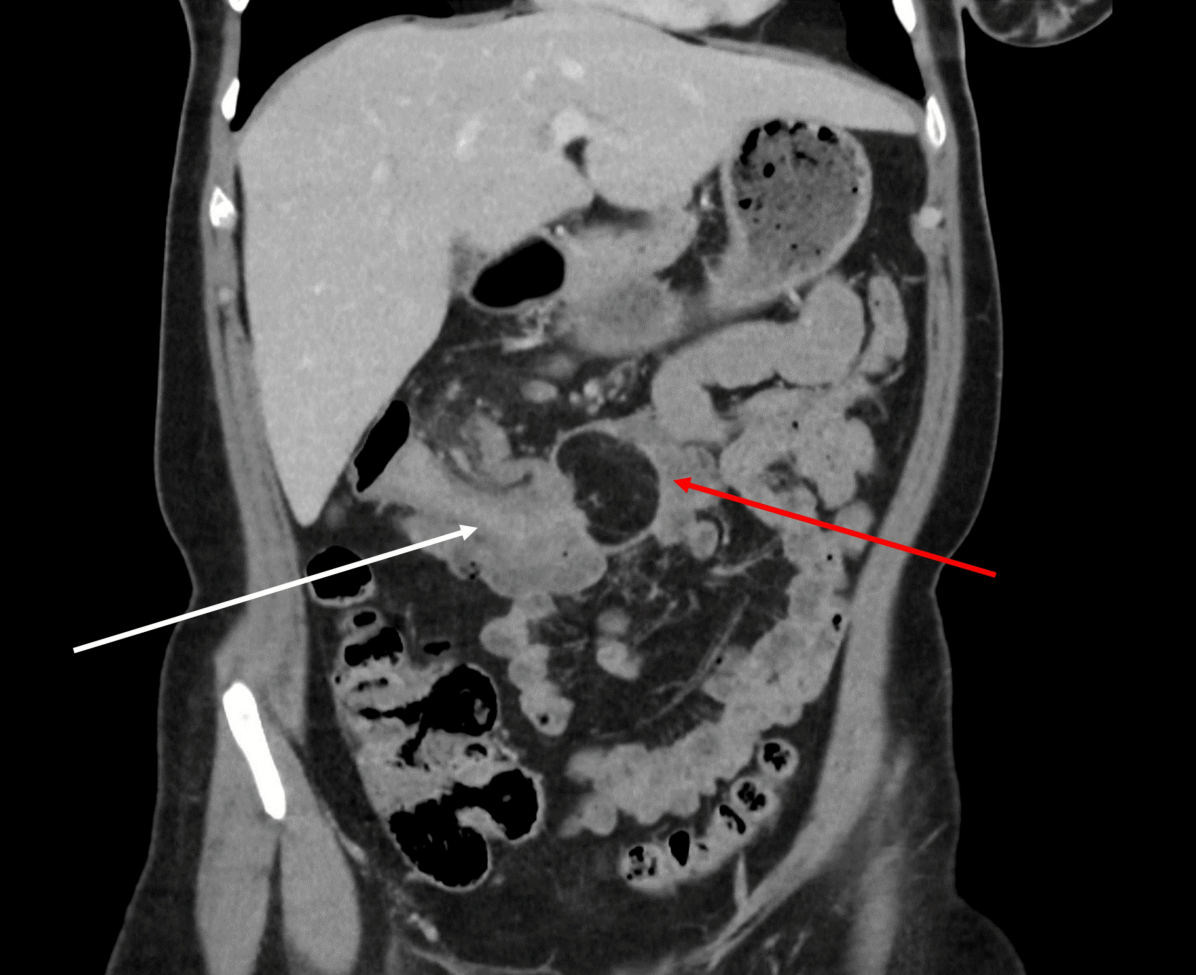
Coronal slice of CT scan in the portal venous phase, with the red arrowing indicating the site of the lipoma, and the white arrow indicating the site of intussusception

**Figure 4:**
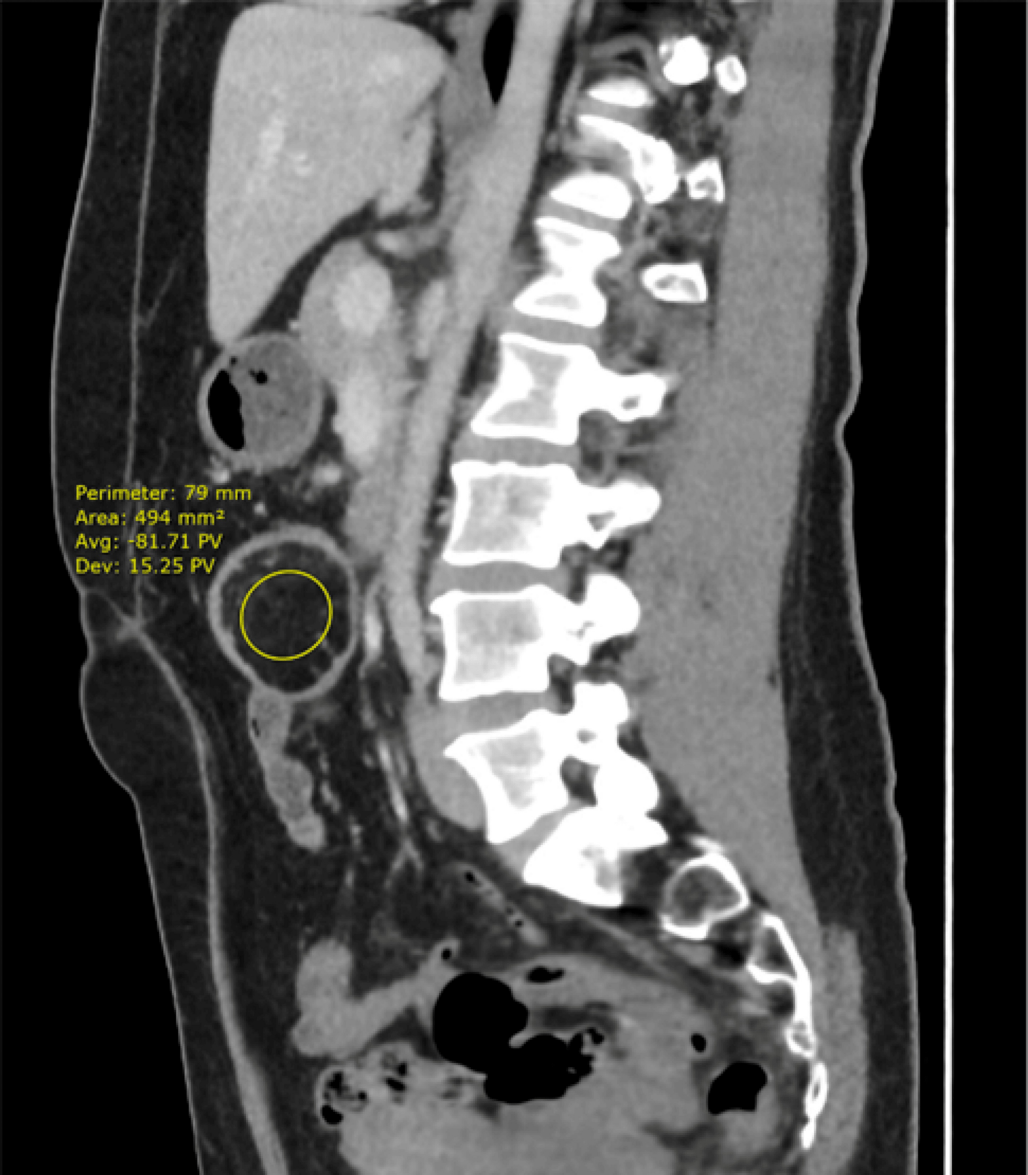
Sagittal view of the CT scan in the portal venous phase showing lipoma as the lead point for colo-colic intussusception HU: Hounsfield Unit The average HU density of the lipoma is shown as -81.

A subsequent CT scan confirmed a colocolic intussusception in the transverse colon with a fat density lesion at the lead point, measuring 34 x 39 mm in the axial plane and 44 mm craniocaudally. The density of the lesion on CT imaging suggested a lipoma, as it had an average Hounsfield Unit (HU) of -81, within the range of -50 to -100 expected for a lipoma [1]. This was later confirmed by histological examination. See Figures 2-4.

The diagnosis of colocolic intussusception, secondary to a transverse colon lipoma, was made based on imaging findings. The patient was initially managed conservatively with analgesia and antiemetics. However, due to worsening symptoms, including vomiting, diarrhea with blood, and severe abdominal pain, surgical intervention was deemed necessary. The patient underwent an open right hemicolectomy to remove the lipoma and resolve the intussusception.

Postoperatively, the histology of the excision sample revealed a benign submucosal lipoma with focal ischaemic ulceration of the overlying mucosa. The patient recovered well postoperatively and was discharged after five days.

## Discussion

Intussusception is a relatively rare condition in adults, accounting for only 5% of all intussusception cases and 1% of all bowel obstructions [1]. The majority of intussusception cases occur in children, with an incidence rate of approximately 35 to 40 per 100,000 children under the age of one [2]. In adults, intussusception is often associated with a pathological lead point, such as a tumor, polyp, or lipoma [2]. Transient small bowel intussusception without a pathological lead has also been described in the literature; however, this is extremely rare [4].

Colonic lipomas are benign tumors composed of mature adipose tissue and are relatively uncommon, with an estimated prevalence of 0.2% to 4.4% in autopsy studies, with only 25% of patients with colonic lipomas developing symptoms [3]. They are typically asymptomatic and discovered incidentally during imaging or endoscopic procedures. However, larger lipomas, particularly those greater than 2 cm in diameter, can cause symptoms such as abdominal pain, bleeding, or obstruction due to their potential to act as a lead point for intussusception [5]. The majority of lipomas are seen in the colon, accounting for 65-75% of all intestinal lipomas, with small bowel lipomas making up around 20-25% [6].

In this case, the patient's intussusception was caused by a colonic lipoma, which was confirmed through a histological examination. The imaging findings were crucial in diagnosing the condition and planning the appropriate surgical intervention. This case underscores the importance of considering lipomas in the differential diagnosis of adult intussusception and highlights the role of imaging and histology in confirming the diagnosis.

## Conclusions

This case report highlights a rare instance of colonic lipoma causing intussusception in an adult. When a mass with the same density as fatty tissue is identified as the lead point for obstruction, a lipoma should be considered the primary differential diagnosis. Despite their generally benign nature, colonic lipomas can lead to serious complications. Therefore, prompt diagnosis and surgical intervention are essential to achieve a favorable outcome.
